# Interplay between Cruciferous Vegetables and the Gut Microbiome: A Multi-Omic Approach

**DOI:** 10.3390/nu15010042

**Published:** 2022-12-22

**Authors:** John A. Bouranis, Laura M. Beaver, Duo Jiang, Jaewoo Choi, Carmen P. Wong, Edward W. Davis, David E. Williams, Thomas J. Sharpton, Jan F. Stevens, Emily Ho

**Affiliations:** 1 School of Biological and Population Health Sciences, Oregon State University, Corvallis, OR 97331, USA; 2 Linus Pauling Institute, Oregon State University, Corvallis, OR 97331, USA; 3 Department of Statistics, Oregon State University, Corvallis, OR 97331, USA; 4 Center for Quantitative Life Sciences, Oregon State University, Corvallis, OR 97331, USA; 5 Department of Environmental and Molecular Toxicology, Oregon State University, Corvallis, OR 97331, USA; 6 Department of Microbiology, Oregon State University, Corvallis, OR 97331, USA; 7 Department of Pharmaceutical Sciences, Oregon State University, Corvallis, OR 97331, USA

**Keywords:** cruciferous vegetables, phytochemicals, multi-omic integration, microbiome, metabolomics

## Abstract

Brassica vegetables contain a multitude of bioactive compounds that prevent and suppress cancer and promote health. Evidence suggests that the gut microbiome may be essential in the production of these compounds; however, the relationship between specific microbes and the abundance of metabolites produced during cruciferous vegetable digestion are still unclear. We utilized an ex vivo human fecal incubation model with in vitro digested broccoli sprouts (Broc), Brussels sprouts (Brus), a combination of the two vegetables (Combo), or a negative control (NC) to investigate microbial metabolites of cruciferous vegetables. We conducted untargeted metabolomics on the fecal cultures by LC-MS/MS and completed 16S rRNA gene sequencing. We identified 72 microbial genera in our samples, 29 of which were significantly differentially abundant between treatment groups. A total of 4499 metabolomic features were found to be significantly different between treatment groups (q ≤ 0.05, fold change > 2). Chemical enrichment analysis revealed 45 classes of compounds to be significantly enriched by brassicas, including long-chain fatty acids, coumaric acids, and peptides. Multi-block PLS-DA and a filtering method were used to identify microbe–metabolite interactions. We identified 373 metabolites from brassica, which had strong relationships with microbes, such as members of the family Clostridiaceae and genus *Intestinibacter*, that may be microbially derived.

## 1. Introduction

Cruciferous vegetable consumption has been associated with a decreased risk of multiple types of cancers, thus presenting a cost-effective, non-pharmacological approach to cancer prevention through dietary intervention [1,2,3,4,5,6,7,8,9,10,11,12,13,14,15,16,17]. Broccoli sprouts and Brussels sprouts are among the leading cruciferous vegetables under study and contain some similar and some distinct phytochemicals that can activate different but complementary mechanisms to promote health [18,19,20,21]. While the cancer-preventative effects of cruciferous vegetables are typically attributed to glucosinolates and their metabolic products, isothiocyanate metabolites, and indoles, other components of cruciferous vegetables could play a synergistic role in conferring cancer-protective and health-promoting effects. Additionally, the metabolism of phytochemicals from cruciferous vegetables by the gut microbiome could further lead to the production, inactivation, or clearance of bioactive dietary components [22]. The gut microbiome is essential to the production of bioactive compounds from various food sources. For example, with soy isoflavones and pomegranate urolithins, the presence or absence of specific microbial taxa directly dictates which metabolites are produced [23,24,25]. A similar paradigm could be extended to cruciferous vegetables in which the gut microbiome may play an important role in driving the inter-individual metabolism of glucosinolates and isothiocyanates, such as sulforaphane.

Despite evidence pointing towards the gut microbiome as playing a pivotal role in driving inter-individual variation in glucosinolate metabolism, little is known about microbial products of cruciferous vegetables beyond isothiocyanate metabolites and nitriles. While work has been conducted to investigate microbial mechanisms of glucosinolate hydrolysis to isothiocyanate metabolites and nitriles, other downstream products of these metabolic pathways are still greatly unknown [26,27,28]. Previous studies examining microbial metabolism of glucosinolates found that while 100% of parent food-based glucosinolates were degraded, only a fraction of the byproducts were recovered in the form of isothiocyanates and related glucosinolate-derived nitriles [29,30]. These observations suggest the presence of unknown microbial products of glucosinolates. Compounds from cruciferous vegetables are also known to alter microbiome composition and metabolism, suggesting a complex interplay between the microbiome and diet [26,27,28,31,32,33]. Additionally, the microbes responsible for the metabolism of glucosinolates, and other cruciferous vegetable phytochemicals, are still unclear, representing a major gap in knowledge. Many studies have been conducted in vivo, in human and rodent models, to examine the impact of cruciferous vegetable consumption on the gut microbiome; however, these analyses are typically strictly taxonomical and do not examine specific microbe–metabolite relationships [26,31,32,33,34,35,36,37,38,39,40]. Metabolomics databases, such HMDB or METLIN, typically focus on endogenous metabolites (i.e., originating from humans), so metabolomic studies, while valuable, do not provide great insight into the diverse array of microbial- and cruciferous vegetable-derived metabolites that are presumed to be present after cruciferous vegetable consumption [41,42,43,44,45,46,47,48,49,50,51,52,53,54]. We recently reported that the gut microbiome composition could influence the production of glucosinolate-derived nitriles from cruciferous vegetables, showing that the presence or absence of specific microbes can influence the abundance of a single metabolite [22]. Thus, we sought to take an untargeted approach to investigate other phytochemicals from cruciferous vegetables that the gut microbiome could play a role in generating.

To investigate plant- and microbe-derived metabolites of cruciferous vegetable digestion and capture information about the microbiome, we utilized an ex vivo fecal incubation system. Our goals were to (1) understand the impact of cruciferous vegetables on the gut microbiome, (2) describe changes to the digestive metabolome following cruciferous vegetable consumption, and (3) identify relationships between specific members of the gut microbiome and specific metabolites. Through this work, we elucidated specific relationships between the gut microbiome and diet-derived metabolites and generated novel hypotheses through which cruciferous vegetables can promote health and prevent disease. Furthermore, by integrating metabolomics and 16S microbiome data, this work helps to bridge the gap between taxonomy and function by examining relationships between microbes and metabolites, allowing for inference on metabolic niche and function.

## 2. Materials and Methods

### 2.1. Ex Vivo Fecal Incubation Model

Broccoli sprouts and Brussels sprouts were in vitro digested using an oral, gastric, and intestinal phase as previously published [22,55,56,57,58,59,60,61,62,63]. Briefly, salivary amylase was added to simulate the oral phase of digestion, which was followed by a gastric phase where samples were acidified to a pH of 2.5 with hydrochloric acid, and pepsin was added. Then sodium hydroxide was added to neutralize the samples (pH 7), and bile salts, pancreatin, and mucin were added for the intestinal phase of digestion. For fecal bacterial cultivation, a 20% fecal slurry (*w*/*v*) was made from fecal material from 10 healthy volunteers (6 female and 4 male, age 17–51, Lee Biosolutions) and sterile PBS (0.1 M pH 7). A total of 500 µL of the fecal slurry was mixed with 10 mL of Brain Heart Infusion Broth (BHI) with hemin and vitamin K, per the manufacturer’s recommendation, and either 500 µL of filter sterilized in vitro digested broccoli sprouts (Broc), 500 µL of filter sterilized in vitro digested Brussels sprouts (Brus), 500 µL of Broc, and 500 µL of Brus were added (Combo), or negative control in vitro digestion (NC). NC contained reverse osmosis water, equivalent in volume to the water content of broccoli sprouts, and underwent the same in vitro digestion procedure as described above with the same enzymes, chemicals, and equipment. Broc and Brus digests were scaled to be equivalent in concentration to a human consuming ½ cup of broccoli or Brussels sprouts, or in the case of the combination, ½ cup of broccoli sprouts and ½ cup of Brussels sprouts. This combination was included as Broc and Brus contain many similar but also some distinct phytochemicals, and thus by combining the vegetables, we increased the dose and broadened the range of phytochemicals from cruciferous vegetables, which can be achieved in the kitchen as a mixed vegetable dish. Fecal cultures were incubated at 37 °C for 24 h in anaerobic conditions [23]. The fecal culture medium was then vortexed, sampled, and centrifuged (13,000× *g*, 10 min), and supernatants were frozen in liquid nitrogen.

### 2.2. Microbial Sequencing

DNA was isolated from fecal cultures using a QIAamp PowerFecal DNA kit (Qiagen) per the manufacturer’s protocol. Fecal DNA concentration was measured using the Qubit dsDNA HS assay kit (Invitrogen). PCR was used to amplify the 16S rRNA gene at the V4 region and then sequenced using an Illumina MiSeq to produce a sequence library using the Earth Microbiome Project protocol [64]. This approach yielded 300 bp paired-end amplicon sequences at a target sequencing depth of 50,000 reads per sample. The 16S amplicon sequencing was performed at the Center for Quantitative Life Sciences core facilities (Oregon State University) using established methods [65]. Data preprocessing and identification of amplicon sequence variations (ASVs) were conducted using the DADA2 pipeline, as implemented in R (v3.5) [66]. Briefly, reads were first trimmed for read quality and then filtered for expected errors, followed by a merging of paired reads and removal of chimeric ASVs. Taxonomy was assigned using the Silva database v132 with the Naïve Bayesian classifier built into DADA2 [66,67] (Supplemental Table S1).

### 2.3. Microbiome Data Management and Quantification of ASVs

All statistical analysis was conducted in R version 4.1.0 unless otherwise noted. The Benjamini–Hochberg procedure was used for multiple testing corrections, and an adjusted *p*-value of 0.05 was used as the significance cutoff [68]. Unannotated taxonomic assignments at the genus level were assigned placeholder names using the form f_FamilyName_ASV#; this method prevents unknown genera (i.e., “NA”) from being removed during agglomeration. ASVs were first agglomerated at the genus level, reducing the number of genera considered in the analysis from 3557 to 935 due to a high number of unannotated species [69]. In order to remove noise from the dataset, sparse genera, which were those observed fewer than four times in at least 20% of the samples and with a mean relative abundance across all samples less than 0.001%, were filtered out of our data set, which yielded a final dataset of 72 genera. Rarefaction curves using the vegan package (v2.6-2) in R were built on agglomerated and filtered data to ensure all samples were sufficiently sequenced (Supplemental Figure S1) [70]. 

### 2.4. Diversity Analysis and Visualization

The R packages phyloseq (v1.4) and ggplot2 (v3.3.6) were used to visualize and calculate alpha-diversity metrics using the unfiltered, un-agglomerated data [69,71]. Differences in alpha diversity were assessed using the Friedman test for accounting for repeated measures. 

### 2.5. Beta-Diversity Analysis

Beta diversity of agglomerated and filtered data was analyzed using Principal Coordinate Analysis (PCoA) and based on Bray–Curtis distance [69]. Permutation analysis of variance (PERMANOVA) was conducted using the adonis function from the vegan package (v2.6-2) [70].

### 2.6. Differential Abundance Analysis

In order to identify genera that were differentially abundant between treatment groups, we utilized a negative binomial generalized linear mixed-effect model, as implemented by the package lme4 (v1.1-30) [72]. All samples were rarefied to an even depth prior to fitting the model. The abundance of each genus was used as the response variable, with the treatment group as the predictor variable. One model was built for each genus. The Benjamini–Hochberg procedure was used to account for multiple tests [68].

### 2.7. Metabolomic Analysis

Metabolites from the fecal culture medium were extracted (100 μL culture/100 μL ice cold 80:20, *v*/*v*, methanol:water), mixed vigorously, and clarified by centrifugation (13,000× *g* for 10 min). The supernatants were further diluted 1:10 (*v*/*v*) with ice-cold 80:20 methanol:water (*v*/*v*) and transferred to mass spectrometry (MS) vials. Briefly, HPLC was performed on a Shimadzu Nexera system with a phenyl-3 stationary phase column (Inertsil Phenyl-3, 5 µm, 4.6 × 150 mm, GL Sciences) coupled to a quadrupole time-of-flight MS (AB SCIEX TripleTOF 5600), as previously described [73,74]. The samples were randomized, auto-calibration was performed every two samples, and a quality control (QC) sample, composed of a pooled aliquot from each sample, was analyzed every 10 samples. MS/MS information was obtained for all samples using information-dependent acquisition (IDA), while sequential window acquisition of all theoretical spectra (SWATH) was performed only on quality control samples. Spectral data were processed using Progenesis QI (NonLinear Dynamics v2.4). Peak deconvolution for [M + H]^+^, [M + Na]^+^, and [M + NH_4_]^+^ adducts in positive ionization mode, and [M − H]^−^, [M + FA − H]^−^, and [M − H_2_O − H]^−^ in negative ionization mode was performed in Progenesis QI. Feature intensities were normalized in Progenesis QI across samples by the total ion current of all features. In order to remove features with high technical variation, features with a CV greater than 50 in the QC samples were removed from the data resulting in 3903 compounds being removed from negative ionization mode and 7355 compounds being removed from positive ionization mode. Additionally, to identify biologically relevant features, features were filtered to only those with a fold-change greater than 2 between treatment groups. The resulting data matrix was exported as a CSV for downstream analysis in R.

### 2.8. Statistical Methods Summary

To summarize, changes to the gut microbiome were evaluated using a mixture of non-parametric and parametric statistical tests to evaluate the effects of cruciferous vegetables on both individual microbes and overall community structure (alpha- and beta-diversity). Changes to the digestive metabolome were evaluated using metabolomics and subsequent generalized linear mixed models, repeated measures ANOVAs, and pairwise t-tests to differentiate between the various treatment groups. Chemical similarity enrichment analyses were used to examine changes to classes of metabolites with exposure to cruciferous vegetables. To find interactions and relationships between the gut microbiome and digestive metabolome (multi-omic integration), a variety of methods were used and described in detail below. Briefly, to identify metabolites and microbes associated with different cruciferous vegetables, a multi-block PLS-DA was used. A correlation circle was used to identify specific pairs of gut microbes and metabolites to be evaluated for correlations that could influence cruciferous vegetable phytochemical metabolism. Next, a data filtering method was used to focus on potential microbial-derived metabolites of cruciferous vegetables, and pairwise relationships between microbes and metabolites were evaluated using Spearman’s rank correlation coefficient, indicating potential relationships between microbes and metabolites. The Spearman’s rank correlations results were presented using a heatmap that clustered metabolites and microbes based on the similarity of their correlations.

### 2.9. Statistical Analysis of Metabolomics Data

In order to determine the impact of treatment on the metabolomic profile of the fecal cultures, repeated measures ANOVA (RMANOVA) was conducted using Progenesis QI software as previously published [41]. The False Discovery Rate (FDR) was controlled at 5% (significance was determined as q < 0.05). Pairwise t-tests, as conducted in Progenesis QI software, were used to find features significantly different between treatment groups. The hyperbolic arcsine (arcsinh) transformation was used prior to all statistical tests being conducted in Progenesis QI.

To find features significantly different between the two microbial sub-populations previously identified in our fecal cultures, we used a generalized linear mixed model (GLMM). In order to handle the large range of values in our metabolomics dataset, log transformation followed by Pareto scaling was applied to the data. Pareto scaling reduces the relative importance of large values while keeping the data structure partially intact [75]. Since Pareto scaling is sensitive to large fold-changes in the data, log2 transformation was first applied. In order to handle 0s in the data, a generalized log transformation was applied following the formula: $\log_{2}x=\frac{\log_{2}\sqrt{x^{2}+a^{2}}}{2}$, where a is the minimum non-zero value in the dataset. We found that the negative control group had an unequal variance from the other three treatment groups, so a separate model was run for NC from the three vegetable treatments (Broc, Brus, Combo). A generalized linear mixed model, as implemented by the lme4 package (v1.1–30) in R, was built for each feature using chromatographic intensity as the response variable and the interaction of treatment and microbial sub-population as the predictor variables [72]. For the NC model, only microbial sub-population was used as the predictor variable. Contrasts, as implemented by the package LmerTest (v3.1–3), were utilized to find features that differed in abundance between microbial sub-populations by treatment group [76]. Multiple hypothesis testing was corrected using the Benjamini–Hochberg method [68]. Statistical results are shown in Supplemental Table S2.

### 2.10. Metabolomics Data Annotation

Data were analyzed with Progenesis QI (Waters Corporation, Newcastle, UK) and PeakView with XIC Manager 1.2.0 (AB SCIEX, Framingham, MA, USA) software. Level 1 and level 2 metabolite annotations were assigned based on the level of confidence of annotations as described [77,78,79]. Level 1 annotations were determined using PeakView by matching accurate mass (error  <  5 ppm), retention time (error  <  10%), MS/MS fragmentation (library score  >  70), and isotope distribution (error  <  20%) with an in-house library of 650 commercially available standards (including IROA Technology, Bolton, MA, USA). These metabolites were then integrated into the Progenesis QI software, where additional Level 2 metabolite annotations were determined using Progenesis QI software and METLIN, Human Metabolome Database, ChemSpider, and MONA libraries [77]. The current data were evaluated based on accurate mass similarity (ppm ≤ 7), isotope similarity (≥70), and fragmentation score (score ≥ 45). Supporting information (Supplemental Table S3) lists identified and putatively assigned metabolites and provides access to the following properties: molecular formula, retention time, monoisotopic ion mass, adducts, mass error library source for identification, and PubChem ID (PCID). Normalized abundances for all features were exported from Progenesis and used for further analysis. To aid in identifying unknown compounds, we utilized de novo annotation techniques and feature-based molecular networking, as implemented by Canopus and Global Natural Products Social Networking, respectively [80,81,82,83,84,85,86,87]. Sirius, a preliminary step to Canopus, was set to the default settings.

### 2.11. Chemical Similarity Enrichment Analysis

To evaluate which classes of compounds were enriched by cruciferous vegetables, we performed Chemical Similarity Enrichment Analysis (ChemRICH) [88]. De novo predicted classes by Canopus were used as grouping information, and *p*-values from the metabolomics pairwise t-tests between indicated treatment groups were used. ChemRICH can only compare 2 groups at a time, so the analysis was run three times to individually compare each cruciferous vegetable treatment group (Broc, Brus, Combo) against NC.

### 2.12. Multi-Block PLS-DA

To integrate metabolomics and microbiome data and identify metabolomic features and microbes associated with cruciferous vegetable consumption, we utilized a multi-block partial least squares discriminant analysis (PLS-DA) [89]. To overcome the high dimensionality of our metabolomics data, we first filtered the dataset only to include features that were found to be significant by RMANOVA or GLMM. In order to simplify the analysis, positive and negative ion metabolomics data were combined utilizing the R package MSCombine (v1.4) [90]. A maximum residual of 0.2 was used as a cutoff value, and the adducts [M + H], [M + NH4], [M + Na], [M–H2O–H], [M–CH2–H], [M–H], [M + Cl], and [M + FA–H] were searched, and the feature in the polarity with a greater intensity was kept. A centered log-ratio (clr) transformation was applied to the microbiome data to center and scale it. Zero-count cells were imputed to 1 prior to the clr-transformation.

The multi-block PLS-DA was conducted as implemented in the R package MixOmics (v6.20.0) [89,91]. Prior to analysis, a multi-level approach to separate within-subject variation from between-subject variation was used to handle the repeated-measures aspect of our study [92]. A design matrix was created with all blocks connected to one another, and a dummy matrix representing the treatment groups was used as the response. The model was built on 8 components, where 15 genera were kept on the first 2 components, with 72 kept on the rest, and 100 metabolomic features were kept on the first two components, with 6062 on the rest. The number of features to keep and the number of components was tuned using a grid search with 10-fold cross validation for each parameter. Centroid distance was used to compare performance between models. Due to general similarity in model performance between the numbers of features kept, a larger model was selected to capture more features at the risk of finding spurious results. Variables were extracted on the first and second components, and an importance cutoff of 0.1 was used. Responder operating characteristic (ROC) curves were constructed using R package pROC (v1.18.0) following leave-one-out cross-validation [93].

### 2.13. Microbial Metabolite Identification

To explore possible microbial-derived metabolites of cruciferous vegetables, we imported data from Progenesis into R and utilized a metabolite filtering approach. Features were filtered down to only those metabolites that were not present in media (mean intensity < 5), the in vitro digested vegetables (mean intensity < 14), and the NC samples (mean intensity < 18), thus removing artifacts of the ex vivo incubation system, incomplete breakdown products of in vitro digestion, and possible microbial-derived metabolites not made from cruciferous vegetables, respectively. To isolate metabolites produced from cruciferous vegetables by bacteria, we further filtered for only compounds with a mean abundance twice as high in Broc, Brus, or Combo as compared to the NC samples. Lastly, we used a Friedman test (non-parametric repeated measures ANOVA) with a Nemenyi post hoc test to identify metabolites that were significantly different between the treatment groups (Broc, Brus, Combo, NC). A correlation analysis was conducted using Spearman’s correlation.

### 2.14. Data and Code Availability

R code containing statistical analyses conducted is available at github under “bouranij/Bouranis_2022_Brassicas_Microbes_Metabolomics”. Raw 16S reads are available in the NCBI SRA under BioProject PRJNA895102. Metabolomics data were submitted to Metabolomics Workbench under ST002338.

## 3. Results

### 3.1. Impact of Cruciferous Vegetables on Microbiome Composition

**Figure 1:**
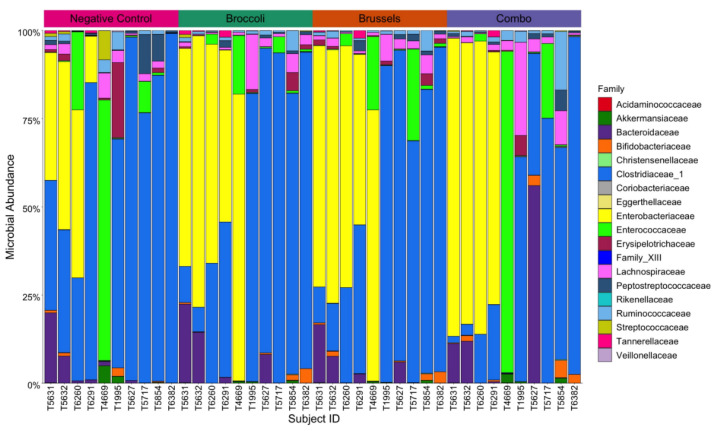
Alpha diversity is not impacted by incubation with in vitro digested cruciferous vegetables. Relative abundance of each family within the fecal culture (*n* = 10). Each treatment group is listed above, and the color and size of each bar correspond to a different family and its relative abundance within each sample.

**Figure 2:**
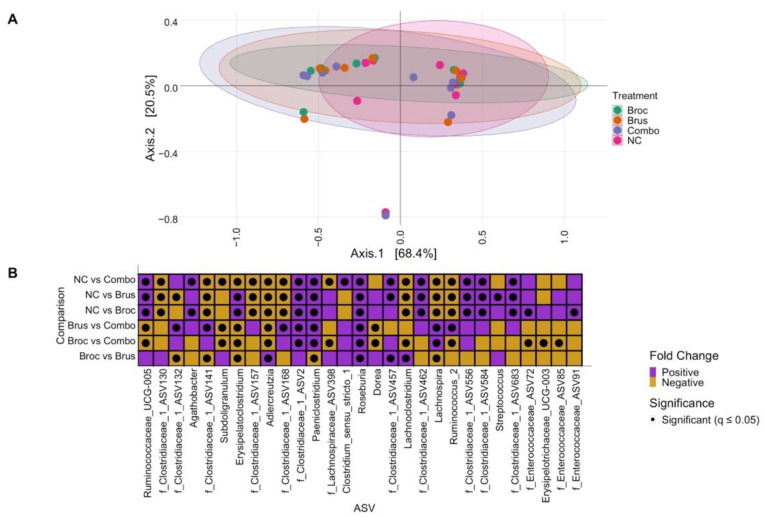
Cruciferous vegetables do not alter β-diversity but do impact individual ASVs. (**A**) Principal coordinates analysis (PCoA) showing beta diversity of samples. Each point represents one sample, with the color of the point indicating the treatment group. Ellipses represent the multivariate t-distribution of each treatment group. (**B**) ASVs significantly different between treatment groups, as determined using a negative binomial generalized linear mixed model. The x-axis represents each genus, and the y-axis represents each pairwise comparison. f_ASV# indicates an unannotated genus of the family shown. Comparisons are organized as Group 1 vs. Group 2; purple squares represent a positive log_2_ fold change, indicating Group 1 is more abundant than Group 2, while orange squares represent a negative fold change, indicating Group 2 is more abundant than Group 1. Black dots within the squares signify significant (q ≤ 0.05) differential abundance.

In total, 72 genera were identified in our samples. In order to determine the overall microbial composition of each sample, alpha-diversity was measured using richness, Simpson, and Shannon indexes, and no significant differences were found between treatment groups for any measures (*p* = 0.782 for richness, *p* = 0.782 for Simpson, *p* = 0.43 for Shannon, respectively) (Figure 1). To evaluate if cruciferous vegetables significantly altered the composition of the gut microbiome and evaluate between-sample compositional differences, we analyzed beta diversity using a principal coordinates analysis (PCoA) (Figure 2A). We did not observe clustering by treatment, suggesting that beta diversity was not altered by cruciferous vegetable. PERMANOVA by treatment was found to be non-significant (*p* = 0.719), validating our observation.

A negative binomial mixed model was used to investigate the impacts of Broc, Brus, and Combo on individual bacterial taxa. Overall, 29 genera were found to be significantly different in their abundance between at least two treatment groups (Figure 2B, Supplemental Table S4). Twenty-six genera were significantly different between NC and at least one cruciferous vegetable group (Broc, Brus, and/or Combo). Of these 26 genera, 11 were found to be significantly more abundant, including four unannotated genera of Clostridiaceae, *Subdoligranulum*, and *Lachnoclostridium*, following incubation with cruciferous vegetables. Fourteen genera were found to be significantly less abundant, including *Agathobacter, Roseburia, Paeniclostridium,* and six unannotated genera of Clostridiaceae, and one was mixed in the direction of the cruciferous vegetable effect depending on the treatment condition (Supplemental Table S4). In order to evaluate the effects of combining cruciferous vegetables compared to a single vegetable, comparisons were made between the Broc and Brus treatment groups and the Combo treatment group. A total of 18 genera were found to be significantly differentially abundant, 10 of which had increased abundance in the Combo group, while seven were significantly less abundant in Combo compared to the single vegetables (Broc and/or Brus, Figure 2B). Lastly, the abundance of nine taxa was found to be significantly different between Broc and Brus treatments (Supplemental Table S4). All significantly different taxa belonged to families Ruminococcaceae, Clostridiaceae, Lachnospiraceae, Erysipelotrichaceae, Eggerthellaceae, Peptostreptococcaceae, Streptococcaceae, and Enterococcaceae.

We had previously identified two sub-populations within our sample donors, those which had microbiomes enriched with bacteria from the family Clostridiaceae and those with bacteria enriched with bacteria from the family Enterobacteriaceae. In this study, these two subpopulations persisted in our dataset, and clear clustering was observed in the PCoA (Figure 2A). PERMANOVA analysis verified the presence of these two bacterial clusters (*p* = 0.001). Cumulatively, while the abundance of individual genera appears to be altered by cruciferous vegetables, there is no effect by in vitro digested Broc, Brus, nor Combo on alpha- nor beta-diversity and the underlying microbial subpopulations (i.e., Clostridiaceae- and Enterobacteriaceae-dominant groups) were not disrupted.

### 3.2. Cruciferous Vegetable Consumption Alters the Digestive Metabolome

In order to describe the impacts of cruciferous vegetables on the digestive metabolome, we conducted an untargeted metabolomics analysis on the fecal cultures. A total of 11,258 features were detected, with 2151 and 2348 features significantly different in negative and positive ionization mode, respectively, when abundances were compared over all four treatment groups (RMANOVA, q ≤ 0.05). Pairwise t-tests delineated which features significantly differed between the specific treatment groups (Supplemental Table S2): 1790 features were different between NC and Broc, 1757 features were different between NC and Brus, and 4545 features were different between NC and Combo. Comparisons between cruciferous vegetable treatments showed 15 features were significantly different between Broc and Brus treatments, while 114 and 139 features were significantly different between Broc and Combo and Brus and Combo, respectively. Principal component analysis (PCA) was conducted on the data, and no separation was observed between any of the treatment groups (Supplemental Figure S2).

**Figure 3:**
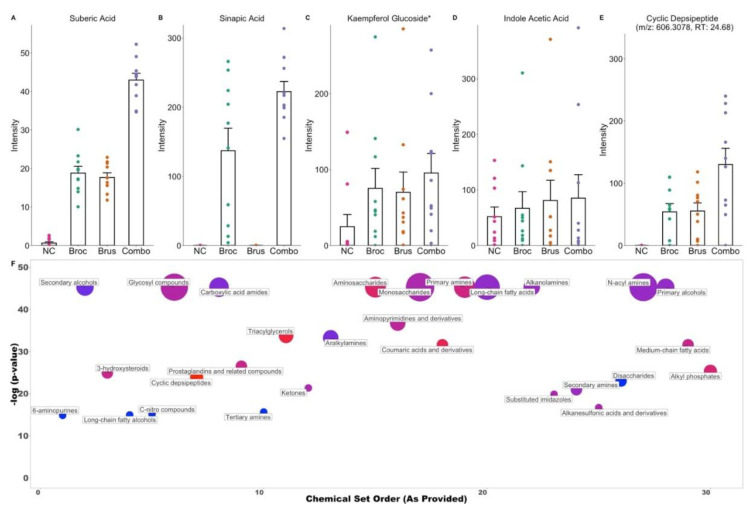
Cruciferous vegetable exposure leads to an increase in plant- and microbial-derived metabolites: (**A**–**E**) Intensity of metabolites in each group, as detected by LC-MS/MS. Each point represents an individual, bars represent means, and error bars show standard error. Metabolite identity of (**A**–**D**) confirmed via MS/MS fragmentation pattern matching. (**E**) Predicted via de novo annotation. (**F**) Chemical Enrichment Analysis (ChemRICH) impact plot for NC vs. Combo. The size of each cluster represents the number of metabolites belonging to it, and the y-axis shows the level of significance; the color of the dot refers to if the compounds were increased (red) or decreased (blue) in Combo relative to NC. Only significant clusters are shown, and peptides and amino acids have been removed for clarity.

Among the features detected, 293 were annotated (Supplemental Table S3), and many were found to be di- and oligo-peptides, most likely incomplete breakdown products from in vitro digestion. Many known plant-derived metabolites were identified, including azelaic acid, suberic acid, sinapic acid, kaempferol glucosides, and 3-formyl indole (Figure 3A–C, Supplemental Tables S2 and S3). Additionally, we identified known microbial-derived metabolites, including indole acetic acid (Figure 3D). Due to the low coverage of plant- and microbe-derived metabolites in available databases, de novo identification methods using Sirius, CSI:FingerID, and Canopus were utilized to elucidate compound class (Supplemental Table S2, Figure 3F). To evaluate which classes of the compound were enriched by cruciferous vegetables, we performed Chemical Similarity Enrichment Analysis (ChemRICH) (Figure 3F). Overall, we found 35 classes of compounds to be significantly enriched by Broc, 36 to be significantly enriched by Brus, and 45 to be significantly enriched by Combo (Supplemental Table S5). Of particular interest, we found long-chain fatty acids and cyclic depsipeptides (Figure 3E,F) to be enriched in all three treatment groups. Additionally, we found coumaric acids and derivatives to be significantly enriched in both Broc and Combo treatments but not in Brus.

We previously identified differential metabolic capabilities in two sub-populations, individuals enriched with Clostridiaceae vs. Enterobacteriaceae, in our data [22]. We next investigated if these two microbial sub-populations were associated with differing digestive metabolomes following cruciferous vegetable consumption and found 534 metabolomic features to be significantly different (Supplemental Table S2). De novo annotation predicted the classes of some of these compounds to be alkanesulfonic acids, benzenesulfonamides, organic sulfuric acids, and thiazoles, all of which contain sulfur atoms (Supplemental Table S2). 

### 3.3. Interplay between Cruciferous Vegetables and the Gut Microbiome

**Figure 4:**
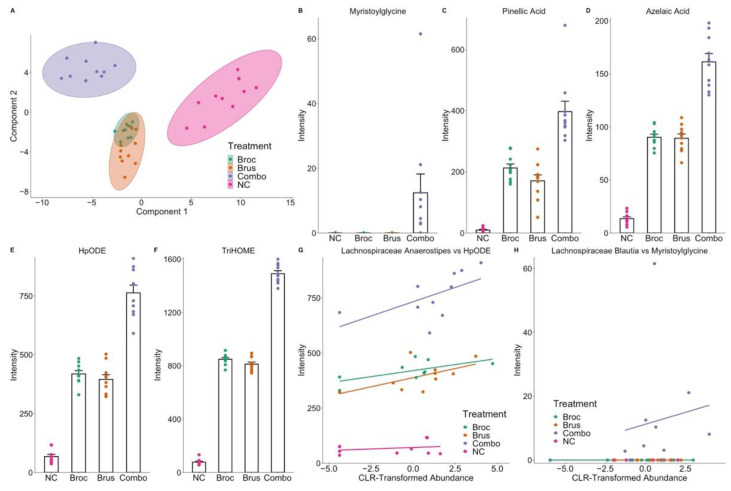
Multi-block PLS-DA reveals signatures of cruciferous vegetable exposure and relationships between specific microbes and metabolites. (**A**) Consensus plot from multi-block PLS-DA analysis. Each point represents one fecal culture, and the color of each point represents the treatment group. (**B**–**F**) Intensity of select metabolites identified by multi-block PLS-DA. Each point represents an individual, bars represent means, and error bars show standard error. (**G**,**H**) Plots comparing intensity of metabolites with the centered log-ratio transformed abundance of select microbes. Each point represents a fecal culture, and lines show within-treatment group correlation. For both presented metabolites, different within-treatment correlations are observed, suggesting that the metabolism of these compounds by the gut microbiome is dependent on the presence of precursor compounds that come from the vegetables themselves.

Having observed changes to both the digestive metabolome and the gut microbiome, we were next interested in finding specific relationships between microbes and metabolomic features using a multi-block partial least squares discriminate analysis (PLS-DA). A discriminate analysis identifies features (i.e., metabolites and microbes), which most separate groups and thus are most associated with each treatment group A consensus plot of individuals, showed clear separation of NC and Combo while Broc and Brus were not able to separate, indicating poor discriminatory power between Broc and Brus (Figure 4A). Nevertheless, sixty-four metabolomic features had importance greater than 0.1, indicating that the metabolite discriminated between at least 2 of the treatment groups (e.g., NC vs. Combo, NC vs. Broc, etc.). Thirty-seven of these sixty-four metabolites were discriminatory towards Combo, twenty-four towards NC, one towards Broc, one towards Brus, and one metabolite was a tie between two groups. These results indicate that there was the greatest discrimination between NC and Combo (Supplemental Table S6). Combo and NC had nine and eight metabolites annotated as peptides, respectively. Three compounds annotated as long-chain fatty acids, a thiazole, a fatty acyl, and an amino fatty acid, were also associated with Combo. Five Combo-associated metabolites were annotated as myristoylglycine, pinellic acid, azelaic acid, HpODE, and TriHOME (Figure 4B–F). Additionally, a benzenoid was associated with Broc. Overall, these classes of compounds could originate from vegetables or from metabolism by the gut microbiome. 

The presence of *Lachnoclostridium, Adlercreutzia*, *Subdoligranulum*, and *Dorea* was associated with Combo. This is consistent with our single-omics results (Supplemental Table S4), where these microbes were observed to have increased abundance in Combo relative to other groups. *Lachnospira*, *Bifidobacterium, Turicibacter*, *Butyricioccus*, and *Faecalibacterium* were all found to be discriminative towards Brus. Interestingly, no microbes were found to be discriminatory towards Broc. Receiver operating characteristic (ROC) curves indicated that the metabolome was a better predictor of treatment class than the microbiome. Additionally, the prediction of NC and Combo is better than the prediction of Broc or Brus (Supplemental Figure S3A).

To further understand the relationship between genera and metabolomic features, a correlation circle was used (Supplemental Figure S3B). Variables that fall further away from the origin (closer to 1) more strongly associate and contribute to a component, while those closer to the origin more weakly contribute. Additionally, variables that are close to one another and thus have an acute angle between them have a positive correlation, while those with an obtuse angle are negatively correlated. Clustering in the feature spaces, using the correlation circle, was used to guide further analysis between specific genera and metabolites. For some features, such as *Anaerostipes* and HpODE, a weak correlation (rho = 0.49) was detected (Figure 4G). For other features, such as Myristoylglycine and *Blautia*, there was no apparent relationship (rho = 0.18) between the metabolite and the genera.

For TriHOME, HpODE, azelaic acid, and pinellic acid, it appears trace amounts could be produced by microbes, as observed by their low abundance in the NC samples, but they are more likely cruciferous vegetable-derived compounds that are metabolized by the gut microbiome (Figure 4B–F). To further investigate this relationship, the correlation circle was used to find genera that are associated with Combo and fall close to HpODE, such as Lachnospiraceae *Anaerostipes* (Supplemental Figure S3B). By using Spearman’s rank correlation, the within-treatment correlation between *Anaerostipes* and HpODE was calculated: rho = 0.517 for Broc, rho = 0.571 for Brus, rho = 0.681 for Combo, and rho = −0.063 for NC (Figure 4G). These results suggest that the presence (or absence) of specific microbial taxa could influence the bioavailability of this compound in vivo. Myristoylglycine, on the other hand, appears to be directly dependent on the combo treatment group, as noted by its absence in the NC, Broc, and Brus treatment groups (Figure 4H). Correlation at the treatment level with an unannotated genus of Lachnospiraceae (represented by ASV354), located closely on the correlation circle (Supplemental Figure S3B), shows a moderate correlation (rho = 0.644) for Combo. Furthermore, this genus is not present in any other treatment groups (other than a single Brus sample), suggesting its growth is dependent on the presence of some factor originating from the combination of Broc and Brus (Combo). It is unclear, however, if the generation of myristoylglycine is dependent on the presence of this microbe due to the low abundance of this compound in some Combo samples, which lack the presence of ASV354. Overall, the multi-block PLSDA revealed relationships between cruciferous vegetables and microbes; however, due to the nature of the analysis, compounds that are solely related to treatment, as opposed to an interaction between the gut microbiome and dietary compounds, also appeared in our analysis.

### 3.4. Identification of Microbial Metabolites of Cruciferous Vegetables

We next examined 387 possible microbial-derived metabolites (detected as described in Section 2.11) that may have been produced from compounds found in broccoli and Brussels sprouts (i.e., not found in NC samples). Many of these metabolites displayed high levels of inter-individual variation, suggesting the gut microbiome played a role in their generation (Supplemental Figure S4). A total of 373 metabolomic features were significantly different between treatment groups (Supplemental Table S7), and post hoc testing revealed that differences between NC and Combo drove the bulk of changes. Many of these compounds were not annotated, so de novo annotation was utilized and predicted triterpenoids, diterpenoids, and flavonoids (Supplemental Table S7), which are of interest as they are typically derived from food sources. Additionally, of 207 metabolites that we could predict a class and chemical formula for, 106 were predicted to contain sulfur atoms, and some were predicted to be azoles, thiodioxopiperazines, peptides, triterpenoids, and benzenoids.

**Figure 5:**
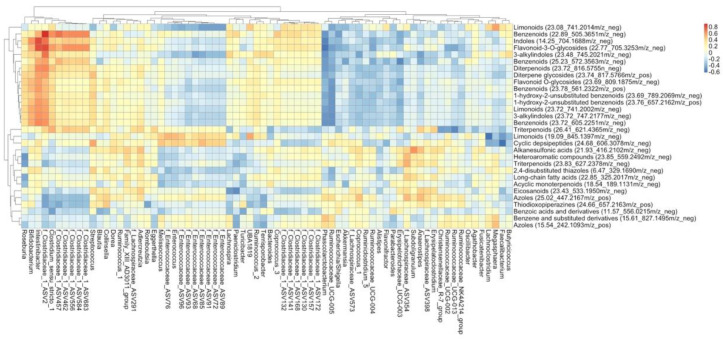
Correlation analysis highlights relationships between specific ASVs and metabolites. Spearman’s correlation of select metabolites with ASVs, tree represents Euclidean clustering of metabolites (y-axis) and ASVs (x-axis).

A correlation analysis was conducted to identify relationships between the identified compounds and microbes (Figure 5.) We observed strong positive correlations between *Intestinibacter*, *Bifidobacterium*, and an unannotated genus of Clostridiaceae (represented by ASV2) and benzenoids, indoles, and flavonoid-3-O-glycosides. Conversely, we detected strong negative correlations with these same compounds and *Phascolarctobacterium* and *Ruminococcaceae UCG-005.* Clustering of bacteria based on correlations with specific metabolites could represent similar metabolic functions despite different taxonomy. For example, the six unannotated genera of Clostridiaceae (represented by ASVs 683, 584, 556, 462, 457, 2) that decreased with cruciferous vegetables, relative to NC, clustered together while the four unannotated genera of Clostridiaceae (represented by ASVs 130, 141, 157, 168), which increased in the presence of cruciferous vegetables, clustered separately. Additionally, for some of these potential microbial metabolites of cruciferous vegetables, these two groups of Clostridiaceae had opposite correlations, suggesting the differential metabolic capabilities of these bacteria.

## 4. Discussion

Overall, in this study, we sought to identify microbial-derived metabolites of cruciferous vegetable phytochemicals and successfully found metabolites of both origins as well as relationships between microbes and metabolites. We observed that the presence of broccoli sprouts and/or Brussels sprouts caused significant changes in the abundance of a variety of individual microbial genera, including members of the families Ruminococcaceae, Clostridiaceae, Lachnospiraceae, Erysipelotrichaceae, Eggerthellaceae, Peptostreptococcaceae, Streptococcaceae, and Enterococcaceae; however, we did not observe changes to alpha- nor beta-diversity. We also showed 45 classes of compounds to be significantly enriched and expected to be in the gut milieu after broccoli and Brussel sprouts were consumed, including long-chain fatty acids, cyclic depsipeptides, coumaric acids and their derivatives, and peptides. Multi-omic integration identified relationships between cruciferous vegetable-derived metabolites myristoylglycine, pinellic acid, azelaic acid, HpODE, and TriHOME and microbes from families Peptostreptococcaceae, Lachnospiraceae, Eggerthellaceae, and Ruminococcaceae. Furthermore, we found 387 microbial-derived metabolites of cruciferous vegetables, many of which displayed high levels of inter-individual variation and correlations with gut microbes. Overall, this study highlights the complexity of microbiome-diet interactions and presents novel findings to be further investigated through mechanistic studies.

Many studies have shown that consumption of various cruciferous vegetables is associated with changes in the microbiome, and our study supports these findings as we found broccoli and Brussels sprouts can affect the abundances of specific microbial taxa [26,28,31,32,33,34,35]. Specifically, we observed changes in bacteria from the families Ruminococcaceae, Lachnospiraceae, Eggerthellaceae, and Streptococcaceae, which have been observed to be altered by cruciferous vegetables in other studies suggesting these bacteria may fill a specific ecological niche that is altered or promoted by compounds in cruciferous vegetables [31,32,33,34,36]. In our study, we observed an increase in four unannotated Clostridiaceae genera and a decrease in seven unannotated Clostridiaceae genera. Previous studies in humans have observed decreases in genus *Clostridium* with consumption of cruciferous vegetables, a result discordant with our findings [31]. One reason for this discrepancy could be because different genera of the family Clostridiaceae respond differently to cruciferous vegetables than those from *Clostridium*. This hypothesis is further supported by examining the correlations between microbial-derived metabolites of cruciferous vegetables, where we observed some unannotated Clostridiaceae genera having strong positive correlations while others had strong negative correlations suggesting differential metabolic processes. Another source of this discrepancy could be the ex vivo nature of our study, where the microbes were exposed solely to the in vitro digested cruciferous vegetables for 24 h as opposed to the study by Kellingray et al., in which humans were fed a cruciferous vegetable-rich diet for 2 weeks, which could have an effect of greater magnitude [31]. 

Additionally, we observed a possible combinatorial effect on the alteration of the gut microbiome when combining two different types of cruciferous vegetables. While broccoli sprouts and Brussels sprouts are similar in composition, they possess different types of glucosinolates, which yield two different classes of hydrolysis products: isothiocyanates, such as sulforaphane, and indoles, respectively. The similar phytochemical composition of these two compounds is reflected by the very low number of significantly different metabolomic features we observed between them. Indeed, it can be argued that the greater amount of plant material in the Combo treatment drove the response; however, evidence in the literature supports the notion that phytochemicals themselves played a role. In Lachnospiraceae *Roseburia*, we observed a decrease in abundance in the Brus and Combo groups, compared to NC; however, the decrease in the Combo group was greater, suggesting a combinatorial effect between Brus and Broc. Previous work has shown that supplementation of indole-3-carbinol (the major hydrolysis product of glucobrassicin in Brussels sprouts) can lead to decreases in bacteria from the family Lachnospiraceae [94]. While we did not detect indole-3-carbinol, the direct hydrolysis product of glucobrassicin in brussels sprouts, we did detect 3-formyl-indole, a bacterial metabolite that is a known agonist for the aryl hydrocarbon receptor (AhR), similar to indole-3-carbinol [95,96]. 

This is the first time, to our knowledge, an untargeted approach has been conducted to characterize the digestive metabolome following the consumption of broccoli sprouts, Brussels sprouts, or their combination. Interestingly, we showed a diverse array of compounds beyond glucosinolate-derived isothiocyanate metabolites and indoles are present in the gut during digestion, many of which have yet to be fully characterized. We utilized an in vitro approach that inherently has limitations, such as failing to capture the role of host dynamics on gut microbiome composition. As metabolomics databases still have a poor annotation of many plant and microbial metabolites, we utilized de novo annotation methods, which predict the class and structure of metabolites based on MS/MS spectra [81,83]. While these methods are informative, they are not as definitive as the use of a standard. Our analysis detected many compounds predicted as benzene and substituted derivatives to be highest in Broc, Brus, and Combo. Another study examining the major constituents of broccoli leaves found benzene-derived compounds, suggesting the compounds we detected are plant metabolites [97]. We also found microbial metabolites of tryptophan, including indole-3-acetic acid and 3-formyl indole, as well as metabolites annotated as terpenoids and triterpenoids. Indole-3-acetic acid has been shown to scavenge free radicals, as well as improve insulin resistance and lipid metabolism in mice [98]. Triterpenoids have been shown in vivo and in vitro to reduce inflammation and have potential in the treatment and prevention of cancer [99,100]. These compounds could offer complementary mechanisms through which cruciferous vegetables promote health and could represent the untapped and unknown potential of microbial-derived metabolites of dietary compounds to improve human health. Future work is needed to elucidate the structure of these metabolites and explore their bioactivity in vivo.

Beyond analyzing the microbiome and metabolome in isolation, we utilized multi-omic integration techniques to find specific relationships between microbes and metabolomic features. The first strategy we used was biologically driven based on previous work from our group, where we observed that the production of sulforaphane nitrile and iberin nitrile were dependent on the composition of the gut microbiome [22]. We observed the same sub-populations in this dataset and identified metabolites that followed a similar paradigm. Next, we used two separate data-driven approaches to highlight bacterial-derived metabolites. Our multi-block PLS-DA highlighted many compounds that appeared to be derived from plants but metabolized by the gut microbiome, potentially impacting their bioavailability in vivo. We also filtered our data based on the abundance of metabolites in each treatment group to select a set of possible bacterial-derived metabolites. De novo annotation predicted many of these metabolites to contain sulfur atoms, which is of interest because cruciferous vegetables contain many sulfurous compounds such as glucosinolates. Additionally, high levels of inter-individual variation were observed in these metabolites between fecal donors suggesting these metabolites are microbially derived. Interestingly, the predicted classes of these metabolites included organonitrogen compounds, benzenoids, alpha amino acids and derivatives, and triterpenoids, all of which are predicted to contain sulfur. These metabolites showed strong correlations with multiple genera, including *Bacteroides*, *Escherichia/Shigella,* and *Akkermansia,* which have been shown to play a role in colonic sulfur metabolism [101]. We also observed strong correlations between these classes of compounds and multiple unannotated genera from the family Clostridiaceae, which we previously showed was associated with the production of nitriles from glucosinolates, possibly through their desulfation [22]. Other studies conducted in humans have shown that cruciferous vegetable consumption leads to a decrease in sulfate-reducing bacteria, while other studies have shown that prolonged consumption of cruciferous vegetables can lead to an increase in the conversion of glucosinolates to isothiocyanate metabolites [26,27,28,31,33,34]. These cumulative findings from across multiple studies point to microbial sulfur metabolism as playing a key role in the metabolism of cruciferous vegetable phytochemicals; however, future work, including metagenomic, proteomic, and other basic-science work must be conducted to identify a causal link between microbial sulfur metabolism and cruciferous vegetable phytochemicals. Additional work can also be completed to validate if the metabolomics results can be reproduced experimentally through in vitro culturing of individual bacteria and subsequent evaluation of the capacity of bacteria of interest to produce the predicted metabolites from cruciferous vegetables.

## Data Availability

R code is available at github under “bouranij/Bouranis_2022_Brassicas_Microbes_Metabolomics”. Raw 16S reads are available in the NCBI SRA under BioProject PRJNA895102. Metabolomics data has been submitted to Metabolomics Workbench under ST002338.

## Supplementary Materials

The following supporting information can be downloaded at: https://www.mdpi.com/article/10.3390/nu15010042/s1. Figure S1: Rarefaction curves of each sample. Each line represents one sample; thus, there is 10 for each treatment group. Each line reaching a plateau indicates that the sample was adequately sequenced. Figure S2: Principal component analysis of metabolomics data following log-transformation and Pareto scaling. Figure S3: (A) Receiver Operator Characteristic (ROC) curves of each component and block for the PLS-DA model following leave-one-out cross validation. (B) Correlation circle of variables with importance greater than 0.1 from the PLS-DA model. Color indicates which group the feature is associated with, and the shape indicates the data block. Figure S4: (A–D) Intensities of some metabolites identified using our filtering method. Each dot represents one sample, and bars represent means. Error bars show standard error. Supplemental Table S1: Taxonomy of ASVs present. Supplemental Table S2: Statistical results, abundances, and de novo annotations of significantly different metabolites. Supplemental Table S3: L1 and L2 annotations of metabolomics data. Supplemental Table S4: Differentially abundant microbes by treatment group. Supplemental Table S5: Chemical enrichment analysis results. Supplemental Table S6: Multi-block PLS-DA group contributions. Supplemental Table S7: Statistical results of microbial-derived metabolites and de novo annotations.
