# Supplementary figures and images for: Interplay between Cruciferous Vegetables and the Gut Microbiome: A Multi-Omic Approach
Source: Nutrients. 2022 Dec 22;15(1):42. doi: 10.3390/nu15010042 (PMC9824405; doi:10.3390/nu15010042)

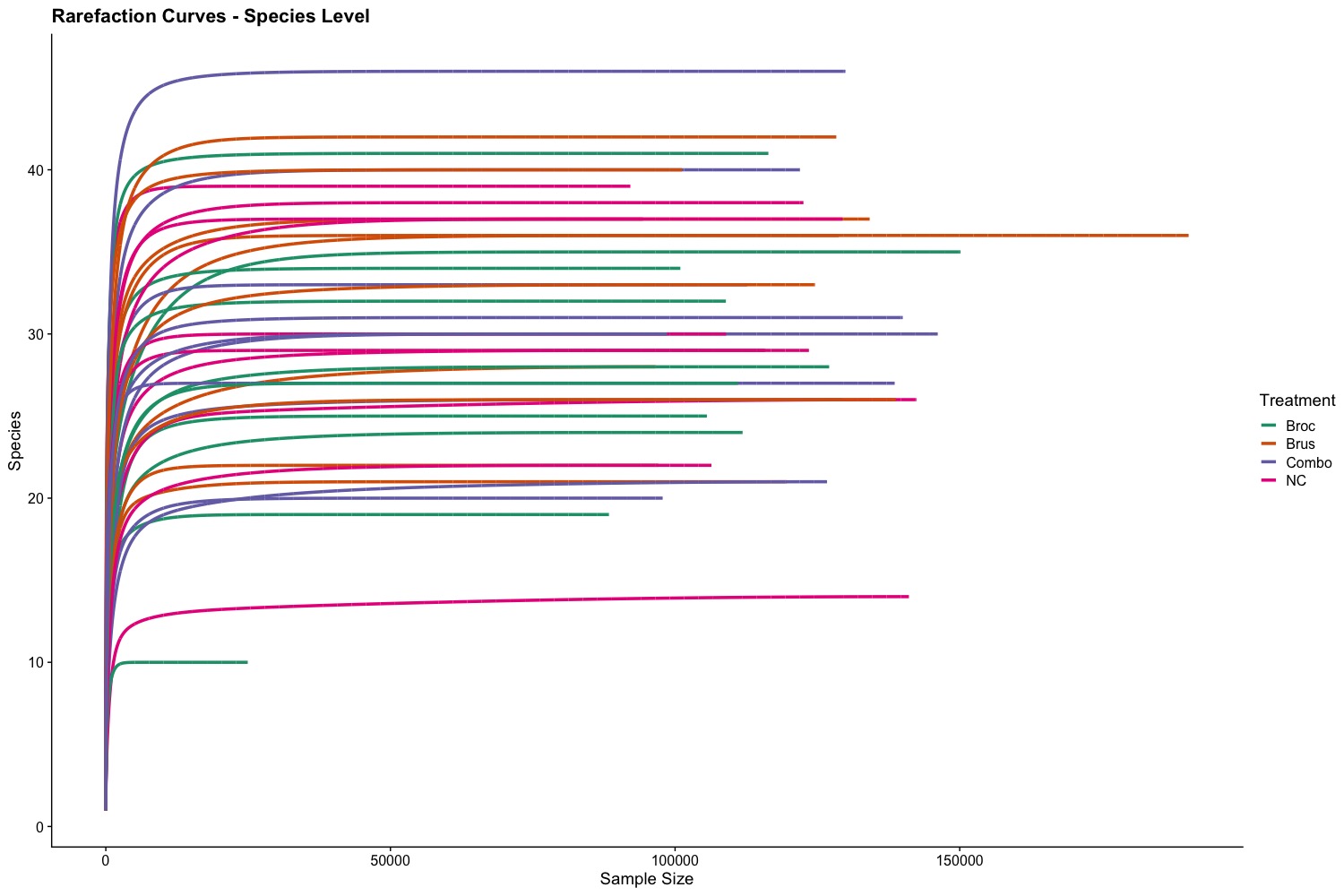

Supplement: Supplementary file 1 [file nutrients-15-00042-s001.zip › Supplemental_Figure_S1.jpeg]

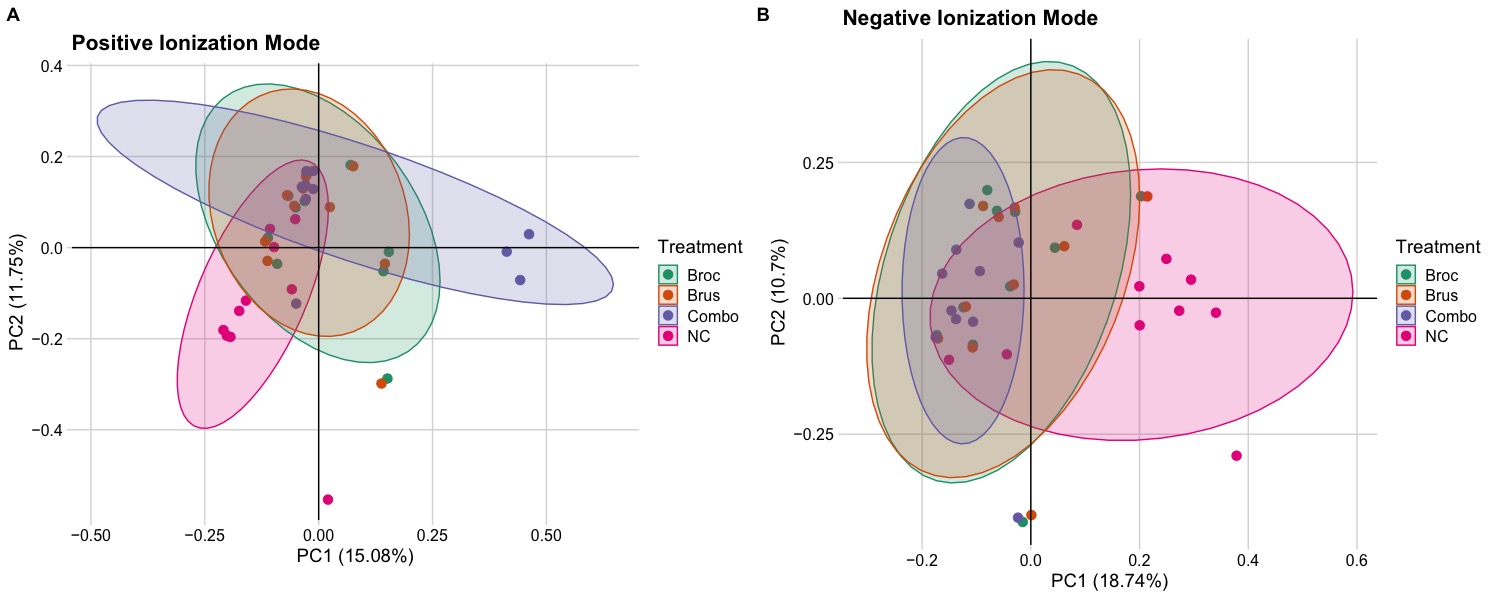

Supplement: Supplementary file 1 [file nutrients-15-00042-s001.zip › Supplemental_Figure_S2_Signs.jpeg]

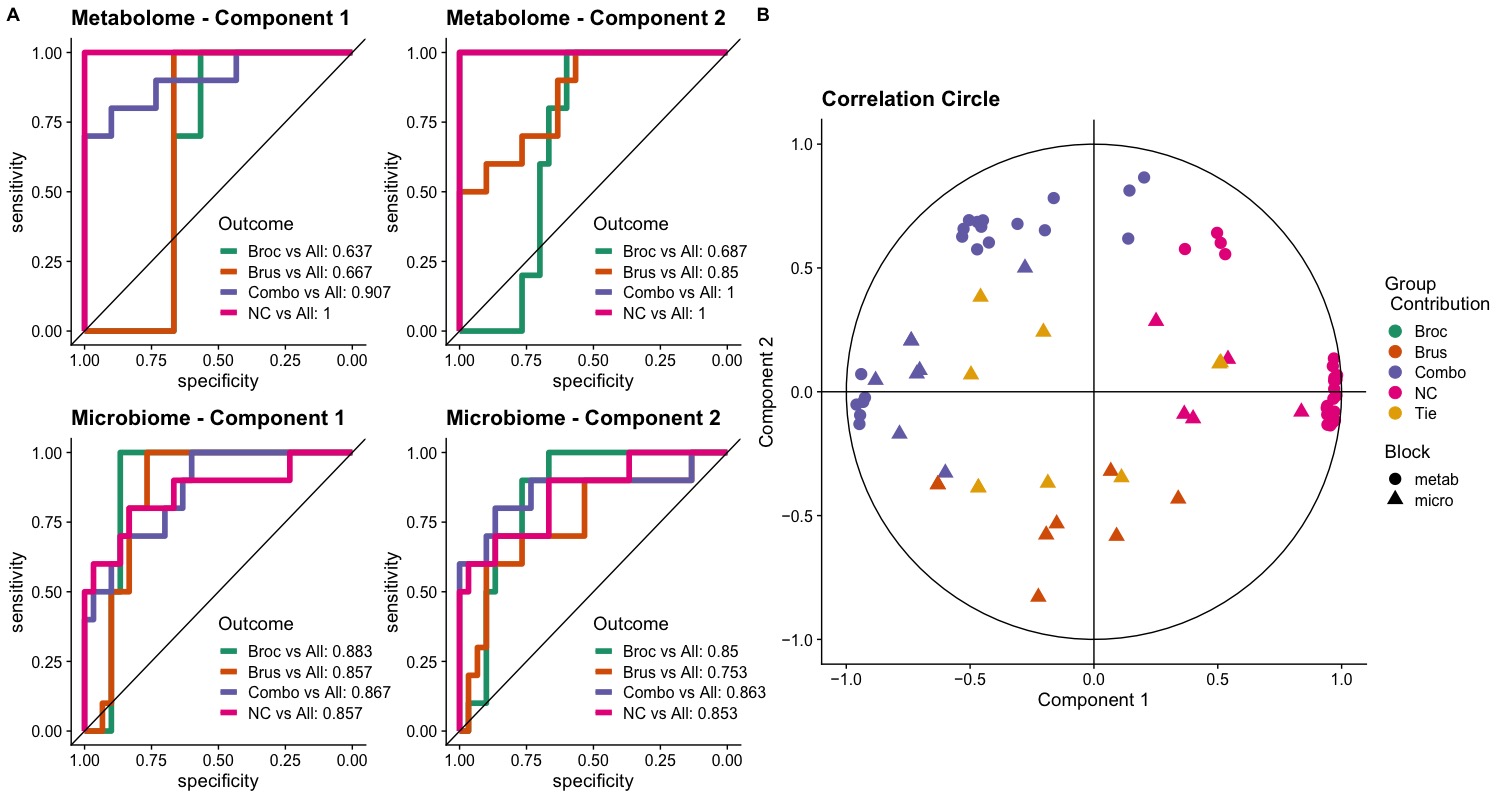

Supplement: Supplementary file 1 [file nutrients-15-00042-s001.zip › Supplemental_Figure_S3_Signs.jpeg]

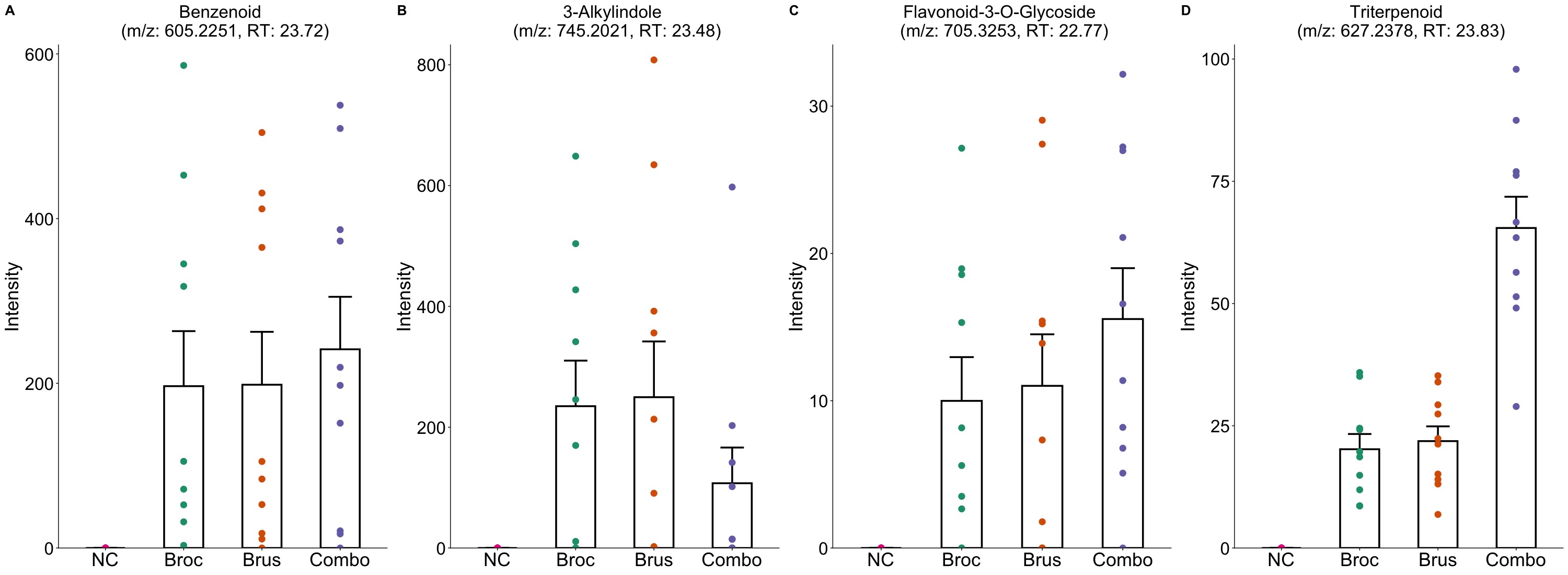

Supplement: Supplementary file 1 [file nutrients-15-00042-s001.zip › Supplemental_Figure_S4.jpeg]
